# TyG index and mild cognitive impairment and dementia: a review of current evidence

**DOI:** 10.3389/fnagi.2026.1825259

**Published:** 2026-07-22

**Authors:** Zhicheng Zhu, Jisheng Xu, Zhiming Tang, Nangen Song

**Affiliations:** 1 Institute of Physical Education, Xinyu University, Xinyu, China; 2 College of Sports Medicine and Health, Chengdu Sports University, Chengdu, China; 3 Sports College, Guangxi College of Sports Education, Nanning, China

**Keywords:** cognition, dementia, insulin resistance, review, TyG

## Abstract

This narrative review aimed to synthesize current epidemiological, mechanistic, and clinical evidence on the association between the triglyceride-glucose (TyG) index and cognitive impairment, and to discuss its potential role in metabolic–neurocognitive risk assessment. Current evidence from cohort studies, cross-sectional analyses, and meta-analyses suggests that higher TyG index levels are generally associated with an increased likelihood of mild cognitive impairment, dementia, and related cognitive decline across different populations. TyG-derived indices that combine the TyG index with anthropometric measures may provide additional information on the combined burden of insulin resistance and adiposity-related metabolic dysfunction. Mechanistically, TyG-related insulin resistance and metabolic dysregulation may be linked to cognitive impairment through multiple interconnected pathways, including impaired cerebral energy metabolism, mitochondrial dysfunction, vascular endothelial dysfunction, blood–brain barrier disruption, abnormal amyloid-β metabolism, tau pathology, and neuroinflammatory responses. From a clinical perspective, the TyG index may be regarded as a simple and accessible metabolic risk-stratification marker, rather than an established diagnostic or predictive biomarker for cognitive impairment. Its potential value may lie in identifying individuals with metabolic dysfunction who may benefit from closer cognitive monitoring and integrated vascular-metabolic risk management. However, because current evidence remains predominantly observational, further prospective, mechanistic, and interventional studies are needed to clarify the causal relevance, incremental predictive value, and clinical utility of the TyG index in cognitive impairment and dementia.

## 1 Introduction

With the acceleration of global population aging, the burden of cognitive impairment and dementia has increased substantially, making them major public health challenges worldwide. According to the World Health Organization, dementia is the seventh leading cause of death globally and one of the leading causes of disability and dependency among older adults. In 2019, an estimated 57 million people were living with dementia worldwide, and this number is projected to rise to 153 million by 2050 (Livingston et al., 2024). Among the various subtypes, Alzheimer’s disease (AD) is the most common form of dementia, accounting for 60%–80% of all cases, while other major types include vascular dementia, dementia with Lewy bodies, and frontotemporal dementia (Podcasy and Epperson, 2016). From a clinical perspective, dementia encompasses stages ranging from mild to severe, whereas mild cognitive impairment (MCI) is generally regarded as an intermediate state between normal cognitive aging and dementia, as well as a prodromal stage and important risk factor for AD and other neurodegenerative disorders (Albert et al., 2011; Ganguli et al., 2013). Patients with dementia often experience progressive deterioration in cognition, behavior, emotion, psychological status, and the ability to perform activities of daily living (Cummings et al., 1994; Lyketsos et al., 2002; McKhann et al., 2011; Arvanitakis et al., 2019). However, by the time memory loss or functional decline becomes clinically apparent, substantial neuronal damage may have already occurred. Evidence suggests that the pathological processes leading to dementia often begin years, or even decades, before the onset of clinical symptoms (Nordberg, 2015). Therefore, early diagnosis of dementia is crucial. It may facilitate timely access to emerging disease-modifying or symptomatic treatments, enable patients to participate in clinical trials, and support informed decisions regarding lifestyle modification, care planning, and future management. In addition, earlier diagnosis may help reduce healthcare costs and improve the quality of life of patients and their families.

However, early diagnosis and risk prediction of dementia remain highly challenging. Conventional diagnosis mainly relies on medical history, neuropsychological assessments, and neuroimaging examinations. Although several imaging approaches have been well validated, including structural magnetic resonance imaging, 18F-fluorodeoxyglucose positron emission tomography, dopaminergic single-photon emission computed tomography, PET imaging for dementia with Lewy bodies, and PET ligand imaging targeting amyloid and tau pathology, their high cost and technical complexity limit their widespread use in community and primary care settings (Arvanitakis et al., 2019; Chouliaras and O’Brien, 2023). Therefore, identifying simple, cost-effective, and easily accessible biomarkers is of considerable importance for the early detection of high-risk individuals, timely intervention, and improved prognosis.

In recent years, increasing attention has been directed toward the link between metabolic dysfunction and neurodegeneration. A growing body of epidemiological and clinical evidence suggests that metabolic syndrome and its central feature, insulin resistance (IR), are closely associated with the onset and progression of cognitive impairment and dementia. IR has been recognized as an important risk factor for the development of neurodegenerative diseases and cognitive decline (Kullmann et al., 2016; Kellar and Craft, 2020; Cui et al., 2022; Kim and Arvanitakis, 2023). Accordingly, biomarkers reflecting IR and metabolic status may have potential value in predicting cognitive deterioration. At present, the hyperinsulinemic-euglycemic clamp is considered the gold standard for assessing IR; however, its invasive nature, high cost, and procedural complexity have limited its widespread use in routine clinical practice (Minh et al., 2021). Similarly, the homeostasis model assessment of insulin resistance (HOMA-IR) has certain limitations, particularly in individuals receiving insulin therapy or those with impaired β-cell function (Tao et al., 2022). In contrast, the triglyceride-glucose (TyG) index has attracted considerable attention because of its simplicity, low cost, and accessibility and has been shown to be associated with the risk of cognitive impairment and dementia. Calculated from fasting triglyceride and fasting blood glucose levels, the TyG index was first proposed by Simental-Mendía et al. in 2008 as a simple surrogate marker of IR (Simental-Mendía et al., 2008). The TyG index is based on the concept that insulin resistance is accompanied by both lipid and glucose metabolic disturbances, manifested as elevated fasting triglyceride and fasting glucose levels. Elevated triglyceride levels may result from impaired insulin-mediated suppression of adipose tissue lipolysis and increased hepatic triglyceride production, while elevated fasting glucose is largely attributable to reduced peripheral glucose uptake and increased hepatic glucose output (Simental-Mendía et al., 2008; Choi and Ginsberg, 2011; Rines et al., 2016; Bjornstad and Eckel, 2018). Because its calculation requires only routine blood test parameters, the TyG index is inexpensive, convenient to obtain, and highly accessible, making it a promising marker for large-scale epidemiological research and preliminary metabolic risk stratification. In the fields of cardiovascular disease and diabetes, the TyG index has demonstrated good predictive performance and has been widely used to identify individuals at high risk of IR and metabolic syndrome (Alizargar et al., 2020; Huang et al., 2022; Huo et al., 2024; Qiao et al., 2025). For example, Cui et al. (2024) reported in a cohort study that the TyG index was significantly associated with MCI in non-diabetic individuals. In addition, Wang H. et al. (2023) demonstrated in a meta-analysis that higher TyG levels were positively associated with the risk of cognitive impairment and dementia and that this association remained significant with each one-unit increase in the TyG index.

Although existing evidence indicates a relationship between the TyG index and cognitive decline, the underlying mechanisms have not yet been fully elucidated, and its clinical value within the biomarker framework of cognitive impairment remains to be further established. Therefore, this review aims to critically summarize current evidence regarding the definition and measurement of the TyG index, its epidemiological association with cognitive impairment, potential biological mechanisms, clinical applicability, and limitations and future research directions. By providing a comprehensive overview of current evidence, this review may help inform future research and support the clinical evaluation of the TyG index in cognitive impairment and dementia.

## 2 Literature search strategy

This article was designed as a narrative review of the current evidence rather than a systematic review or meta-analysis. Nevertheless, a structured literature search was conducted to improve the transparency and reproducibility of the review process. Relevant studies were identified by searching PubMed, Web of Science, Scopus, Embase, and Google Scholar from database inception to June 20, 2026. The search strategy combined terms related to the triglyceride-glucose index with terms related to cognitive impairment and dementia. The following keywords and their combinations were used: “triglyceride-glucose index”, “TyG index”, “triglyceride glucose index”, “insulin resistance”, “mild cognitive impairment”, “MCI”, “cognitive impairment”, “cognitive decline”, “cognitive function”, “dementia”, “Alzheimer’s disease”, and “vascular dementia”. Boolean operators were used to combine search terms. The main search strategy was as follows: (“triglyceride-glucose index” OR “TyG index” OR “triglyceride glucose index”) AND (“mild cognitive impairment” OR “MCI” OR “cognitive impairment” OR “cognitive decline” OR “cognitive function” OR “dementia” OR “Alzheimer’s disease” OR “vascular dementia”).

The search was limited to studies published in English. Observational studies, cohort studies, cross-sectional studies, case-control studies, and relevant review articles were considered eligible if they examined the association between the TyG index and mild cognitive impairment, cognitive decline, dementia, or related cognitive outcomes. Studies focusing on insulin resistance, glucose metabolism, lipid metabolism, vascular injury, neuroinflammation, oxidative stress, blood-brain barrier dysfunction, or Alzheimer’s disease pathology were also reviewed when they provided mechanistic evidence relevant to the potential link between the TyG index and cognitive impairment. Reference lists of included articles and relevant reviews were manually screened to identify additional eligible studies. Studies were excluded if they did not assess the TyG index or its related derivatives, did not report cognitive outcomes, focused exclusively on unrelated metabolic or cardiovascular outcomes without cognitive relevance, or were conference abstracts, editorials, letters, or case reports without original data. The main characteristics and findings of key epidemiological studies identified through this search are summarized in Supplementary Table 1.

## 3 Definition and measurement of the TyG index

The TyG index is a composite indicator calculated from fasting triglyceride and fasting blood glucose levels and is intended to assess the degree of insulin resistance and overall metabolic status in an individual. It is calculated using the following formula: ln [fasting triglycerides (mg/dL) × fasting blood glucose (mg/dL)/2]. Owing to its acceptable diagnostic performance for insulin resistance in validation studies, cost-effectiveness, and the fact that it does not require insulin measurement, the TyG index has been widely used in epidemiological and clinical research (Guerrero-Romero et al., 2010). Compared with conventional approaches that rely on insulin assays, the TyG index provides a simple and economical alternative.

Since its first proposal in 2008, the TyG index has been validated and applied in numerous studies (Simental-Mendía et al., 2008). Simental-Mendía et al. initially proposed this index to identify individuals with insulin resistance using routine fasting triglyceride and glucose parameters. Subsequently, Guerrero-Romero et al. further validated the TyG index against the euglycemic-hyperinsulinemic clamp, the gold standard for assessing insulin sensitivity. They reported a significant inverse correlation between the TyG index and the clamp-derived total glucose metabolism rate (Pearson’s *r* = -0.681, *P* < 0.005). In addition, the TyG index demonstrated good diagnostic performance for insulin resistance, with an optimal cut-off value of 4.68 using the formula adopted in that study, a sensitivity of 96.5%, a specificity of 85.0%, and an area under the receiver operating characteristic curve of 0.858 (Guerrero-Romero et al., 2010). These findings support the reliability of the TyG index as a simple and accessible surrogate marker of insulin resistance. In addition, a large body of clinical evidence suggests that the application of the TyG index extends beyond diabetes to non-diabetic populations and a wide range of cardiovascular conditions, including stable coronary artery disease, acute coronary syndrome, in-stent restenosis, atherosclerosis, coronary artery calcification, and heart failure (Tao et al., 2022). In recent years, its application has been further expanded to non-alcoholic fatty liver disease, kidney disease, and reproductive disorders (Sun et al., 2025). Across these studies, the TyG index has generally demonstrated predictive performance comparable to that of traditional IR indicators, such as HOMA-IR, while offering superior clinical practicality. With increasing attention being paid to metabolic–neural interactions, research on the TyG index in the field of cognitive impairment has also grown steadily, making it an important link between metabolic abnormalities and neurodegeneration.

The TyG index and traditional indicators such as HOMA-IR each have distinct strengths and limitations. HOMA-IR directly reflects the homeostatic relationship between fasting insulin and blood glucose and is widely regarded as a reliable surrogate marker for IR (Tahapary et al., 2022). However, HOMA-IR requires fasting insulin measurement, which is not routinely performed in standard physical examinations, and insulin levels are susceptible to variation due to testing time, physiological stress, and other factors, potentially resulting in considerable measurement variability (Ezaki et al., 2020). By contrast, the TyG index does not require insulin measurement, thereby avoiding the influence of insulin secretion rhythms and assay-related errors and offering better reproducibility. Moreover, by integrating information on both blood lipids and glucose, the TyG index reflects the metabolic characteristics of IR and metabolic syndrome from a more comprehensive perspective. Several studies have directly compared the predictive performance of the TyG index and HOMA-IR for metabolically related diseases and found a good correlation between the two, while the TyG index often shows similar or even superior performance in predicting cardiovascular events and diabetes risk (Vasques et al., 2011; Lee et al., 2014; Zhou et al., 2026). Therefore, in clinical and epidemiological settings, the TyG index may serve as a complementary marker to HOMA-IR for identifying individuals at high risk of IR and metabolic abnormalities.

## 4 Epidemiological evidence linking the TyG index to cognitive impairment and dementia

The main characteristics, study populations, TyG-related exposures, cognitive outcomes, and principal findings of relevant epidemiological studies are summarized in Supplementary Table 1.

Mild cognitive impairment is an intermediate state between normal aging and dementia, characterized primarily by mild decline in memory or other cognitive domains, while activities of daily living remain largely preserved (Langa and Levine, 2014). Increasing evidence suggests that the TyG index is closely associated with the risk of MCI. In the general older population, an elevated TyG index has been reported as an independent risk marker associated with MCI. For example, Feng L. et al. (2025) in a study of 1,163 patients with coronary heart disease, reported that the TyG index was negatively correlated with cognitive test scores and that individuals with a TyG index ≥ 8.72 had a 3.69-fold higher risk of MCI than those with lower TyG values. These findings suggest that, in patients with cardiovascular disease, the TyG index may serve as a useful marker for identifying individuals at high risk of MCI.

The association between the TyG index and MCI has also been observed in patients with type 2 diabetes mellitus (T2DM). A study involving 243 patients with T2DM found that those with coexisting MCI had significantly higher TyG index values than cognitively normal patients with T2DM (Feng S. et al., 2025). Further analyses showed that, among patients with T2DM without diabetic nephropathy, a higher TyG index was significantly associated with poorer global cognitive performance, executive function, and immediate memory. This suggests that an elevated TyG index may be independently associated with MCI in patients with T2DM, particularly in those without severe microvascular complications such as diabetic nephropathy. However, in patients with T2DM complicated by diabetic nephropathy, the association between the TyG index and cognitive function was no longer significant. This may indicate that severe renal impairment either attenuates the cognitive effects of the TyG index or that other factors, such as uremic toxins and anemia, play a more dominant role in cognitive decline in this subgroup. Despite this heterogeneity, the overall evidence supports an association between higher TyG levels and an increased risk of MCI, especially in relation to impairments in executive function and immediate memory. Similar findings were also reported by Tong et al. (2022).

Alzheimer’s disease, the most common type of dementia, has also been increasingly investigated in relation to the TyG index. In a retrospective observational cohort study involving 5,586,048 participants aged 40 years and older, Hong et al. (2021) found that individuals in the highest TyG quartile had a significantly higher risk of AD than those in the lowest quartile. Guan et al. (2025), in a prospective longitudinal cohort study of 2,170 participants free of AD at baseline, further explored the relationship between trajectories of the TyG index and incident AD. Compared with the low-trajectory group, both the moderate- and high-trajectory groups showed a higher risk of developing AD, suggesting that sustained elevation of the TyG index is associated with a higher risk of incident AD. In addition, the TyG index has shown certain associations with AD-related pathological biomarkers. A study of 628 non-demented individuals found that a higher TyG index was significantly associated with plasma Aβ42 and Tau levels, suggesting a potential link between the TyG index and AD-related pathological processes (Zhang et al., 2024b). However, another study reported that the TyG index was linearly associated with elevated plasma Aβ40, Aβ42, and the Aβ42/Aβ40 ratio, but not significantly associated with plasma total tau (t-tau) (Tian et al., 2023). Although these findings require further validation, the available evidence suggests that the TyG index may be associated with AD-related biomarkers to some extent.

In addition to AD, vascular dementia is also closely related to metabolic syndrome and insulin resistance. As a metabolic indicator, the TyG index may theoretically be relevant to vascular dementia risk. However, direct evidence on the relationship between the TyG index and vascular dementia remains limited. Likewise, for other dementia subtypes, such as dementia with Lewy bodies and frontotemporal dementia, direct data are still lacking. Nevertheless, because metabolic factors are also involved in the pathogenesis of these disorders, whether the TyG index plays a role in their development warrants further investigation.

As research on the TyG index has progressed, investigators have increasingly examined TyG-derived indices that combine the TyG index with anthropometric measures to better characterize the joint burden of insulin resistance and adiposity. TyG-derived indices, such as TyG-body mass index (TyG-BMI), TyG-waist circumference (TyG-WC), and TyG-waist-to-height ratio (TyG-WHtR), have attracted increasing attention in studies of metabolic disease and cognitive function. For example, Wei et al. (2025) reported that TyG-BMI, TyG-WC, and TyG-WHtR were all positively associated with motoric cognitive risk syndrome, among which TyG-BMI showed the most stable association. When TyG-BMI was below a certain threshold, the risk of motoric cognitive risk syndrome increased with increasing TyG-BMI. Similar findings were reported by Cui et al. and Tong et al., both of whom showed a significant association between TyG-BMI and MCI (Tong et al., 2022; Cui et al., 2024). In addition, Zhang et al. (Zhang et al., 2024a) demonstrated that TyG-BMI was also associated with AD pathology, cognition, and brain structure in non-demented individuals.

Although TyG-derived indices overlap conceptually with metabolic syndrome, they are not identical to metabolic syndrome as a diagnostic construct. Metabolic syndrome is usually defined as a categorical condition based on the coexistence of several cardiometabolic abnormalities, including abdominal obesity, hypertriglyceridemia, low high-density lipoprotein cholesterol, elevated blood pressure, and impaired fasting glucose (Alberti et al., 2009). By contrast, TyG-BMI, TyG-WC, and TyG-WHtR are continuous indices that combine the TyG index, a surrogate marker of insulin resistance, with anthropometric measures of general or central adiposity (Er et al., 2016; Song et al., 2021; Hao et al., 2024). TyG-BMI mainly reflects the combined burden of insulin resistance and overall adiposity, whereas TyG-WC and TyG-WHtR place greater emphasis on central fat accumulation. In particular, TyG-WHtR adjusts waist circumference for height and may better capture body-shape-related metabolic risk across individuals with different body sizes (Song et al., 2021; Hao et al., 2024).

From this perspective, TyG-derived indices may provide additional information beyond metabolic syndrome by capturing gradations of insulin resistance and adiposity-related metabolic risk rather than relying on fixed diagnostic thresholds. This may be useful in cognitive outcomes research, where subclinical metabolic dysfunction and cumulative metabolic burden may influence cognitive decline before individuals meet formal criteria for metabolic syndrome (Hao et al., 2024; Duan et al., 2026; Wei et al., 2026). However, these indices should be regarded as complementary to, rather than replacements for, metabolic syndrome. Metabolic syndrome remains clinically useful because it incorporates a broader cluster of cardiometabolic abnormalities, including blood pressure and HDL cholesterol, which are not included in TyG-derived indices (Alberti et al., 2009). Therefore, whether TyG-BMI, TyG-WC, or TyG-WHtR provides superior predictive value over metabolic syndrome for cognitive impairment remains to be determined in future studies using direct comparative models.

Overall, current epidemiological evidence suggests that higher TyG levels and TyG-derived indices are associated with an increased risk of cognitive decline across both MCI and dementia stages. Although TyG-derived indices may provide complementary information beyond metabolic syndrome by capturing continuous metabolic and adiposity-related risk, their added predictive value remains to be fully established. Most available studies are cross-sectional or retrospective, and further prospective studies are needed to clarify the temporal relationship, generalizability, and comparative utility of these associations.

## 5 Biological mechanisms linking the TyG index to cognitive impairment and dementia

**FIGURE 1:**
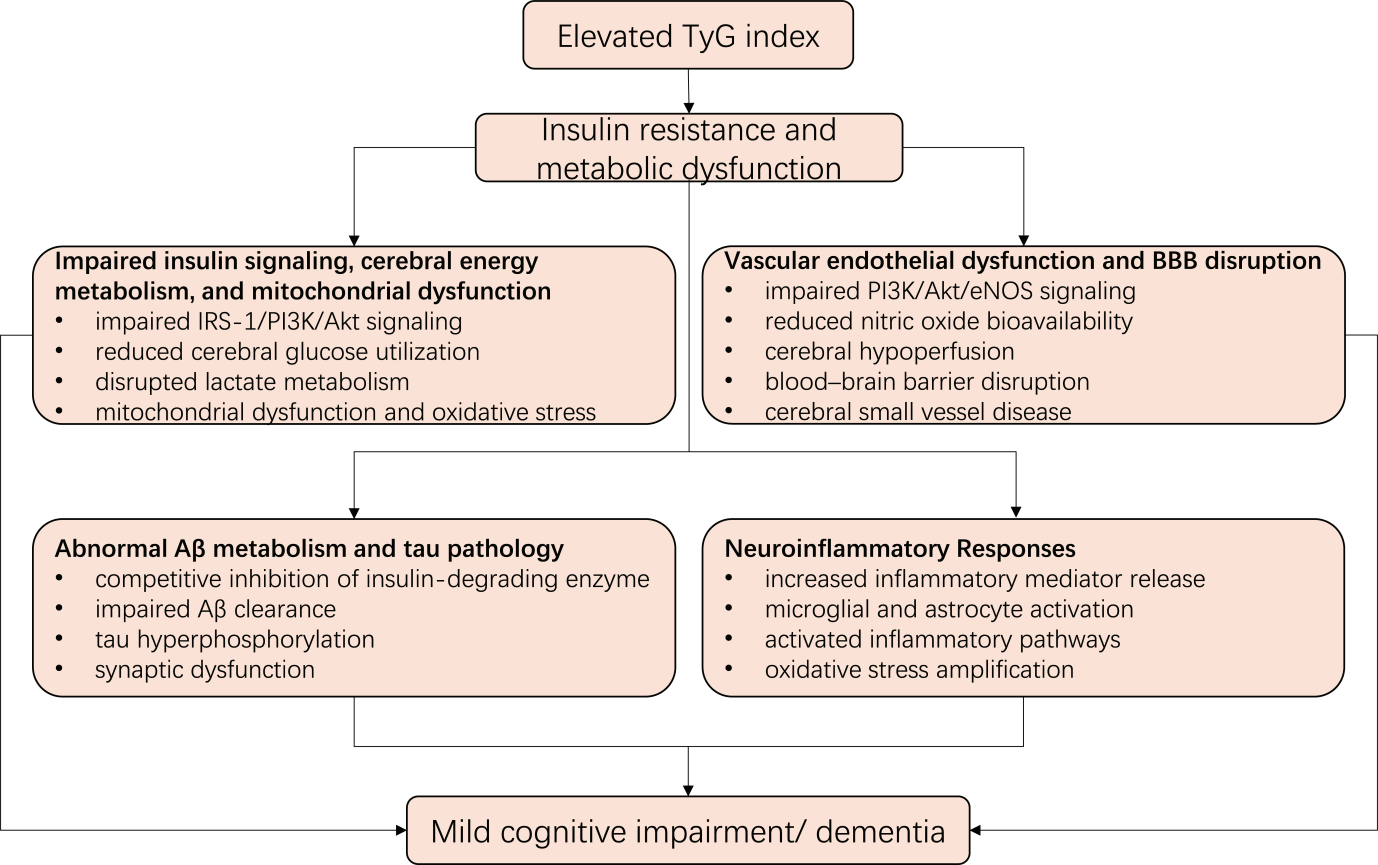
Biological mechanisms linking the TyG index to cognitive impairment and dementia.

Although a substantial body of epidemiological evidence has shown that an elevated TyG index is closely associated with cognitive decline and an increased risk of dementia, the underlying biological mechanisms remain incompletely understood. Current evidence suggests that the insulin resistance and metabolic dysregulation reflected by the TyG index may be associated with changes in brain structure and function through multiple interconnected pathways, thereby potentially contributing to cognitive impairment (Figure 1). These pathways may involve impaired insulin signaling and cerebral energy metabolism, mitochondrial dysfunction, vascular endothelial injury and blood–brain barrier (BBB) disruption, abnormal amyloid-β (Aβ) and tau metabolism, and neuroinflammatory responses.

### 5.1 Impaired insulin signaling, cerebral energy metabolism, and mitochondrial dysfunction

Under conditions of IR, the physiological responsiveness of tissues to insulin is reduced, resulting in disrupted glucose utilization (James et al., 2021). As the brain is a highly energy-demanding organ that relies heavily on glucose as its primary energy source, cerebral energy metabolism is also affected by IR (Lanzillotta et al., 2026). Under normal conditions, insulin facilitates neuronal glucose uptake and utilization, thereby helping maintain neuronal integrity and synaptic plasticity (Arnold et al., 2018). At the molecular level, insulin binding to the insulin receptor activates insulin receptor substrate (IRS)-1/2, phosphatidylinositol 3-kinase (PI3K), and protein kinase B/Akt signaling, which are important for neuronal metabolism, synaptic function, and cell survival (Talbot et al., 2012; Mullins et al., 2017). However, when IR develops, impaired insulin receptor signaling and abnormal IRS phosphorylation may reduce downstream PI3K/Akt activation, thereby weakening insulin-mediated neuroprotective effects (Talbot et al., 2012; Yarchoan et al., 2014).

This disturbance in brain insulin signaling may reduce the ability of neurons to utilize glucose and lead to insufficient cerebral energy supply. Reduced cerebral glucose metabolism may impair ATP production, synaptic plasticity, and neurotransmitter synthesis, thereby compromising cognitive functions such as learning and memory and increasing the risk of neurodegenerative disease (Tramutola et al., 2015).

In addition, IR has been linked to abnormalities in cerebral lactate metabolism. As a highly energy-consuming organ, the normal function of the brain depends on a stable supply of glucose and efficient metabolic regulation (Lundgaard et al., 2015). When IR occurs, this finely regulated energy network may be disrupted, leading to abnormalities in the production, transport, and utilization of lactate, which serves as both an important energy substrate and a signaling molecule. This may further exacerbate neuronal energy deficiency and functional impairment (Zhao et al., 2018).

Insulin resistance may also contribute to mitochondrial dysfunction, which provides an additional link between metabolic disturbance and neurodegeneration. Hyperglycemia, lipotoxicity, oxidative stress, and chronic inflammation can impair mitochondrial oxidative phosphorylation, increase reactive oxygen species production, and disrupt mitochondrial dynamics (De Felice and Ferreira, 2014). These changes may further contribute to neuronal energy stress, synaptic dysfunction, and increased neuronal vulnerability to Aβ toxicity and tau-related injury (De Felice and Ferreira, 2014; Mullins et al., 2017). Therefore, the systemic IR reflected by an elevated TyG index may be associated with cognitive dysfunction, partly through reduced cerebral energy utilization and impaired neuronal metabolic efficiency. Some studies have suggested that pharmacological improvement of insulin sensitivity may be associated with slower cognitive decline in individuals with metabolic dysfunction (Ribarič, 2016; Pelle et al., 2023). Accordingly, impaired cerebral energy metabolism may represent one of the key pathways through which the TyG index influences cognitive function.

### 5.2 Vascular endothelial dysfunction and blood–brain barrier disruption

The BBB is a critical structure for maintaining the stability of the cerebral microenvironment, and its disruption is considered one of the early events in cognitive impairment (Nation et al., 2019). Recent studies have shown that IR is closely associated with BBB dysfunction (Rhea and Banks, 2019). Insulin and insulin-like growth factors play important roles in regulating BBB permeability (Pelle et al., 2023). Under conditions of IR, weakened insulin signaling may reduce the expression of tight junction proteins in BBB endothelial cells, thereby increasing BBB permeability (Zhu et al., 2025).

In addition to BBB injury, IR may also impair vascular endothelial function, which is an important vascular pathway linking metabolic dysfunction to cognitive decline. In endothelial cells, insulin receptor activation normally stimulates the IRS-1/PI3K/Akt/endothelial nitric oxide synthase (eNOS) pathway, leading to nitric oxide (NO) production and vasodilation (Muniyappa and Sowers, 2013). In the insulin-resistant state, this vasodilatory pathway is impaired, resulting in reduced NO bioavailability. Meanwhile, mitogen-activated protein kinase (MAPK)-related signaling and endothelin-1-mediated vasoconstrictive effects may be relatively preserved or enhanced, creating an imbalance between vasodilation and vasoconstriction (Muniyappa and Sowers, 2013). This imbalance promotes endothelial dysfunction, oxidative stress, inflammation, vascular remodeling, and impaired blood flow regulation.

When endothelial dysfunction occurs in the cerebral circulation, it may reduce cerebrovascular reactivity and contribute to cerebral hypoperfusion. Chronic cerebral hypoperfusion can damage neurons and white matter, particularly in regions vulnerable to small vessel injury. IR-related endothelial dysfunction may also promote cerebral small vessel disease, including white matter hyperintensities, lacunar infarcts, microbleeds, and enlarged perivascular spaces, which are closely associated with cognitive decline and vascular cognitive impairment (Iadecola, 2013). Therefore, vascular dysfunction may represent an important mechanism through which the TyG index is related not only to Alzheimer’s disease but also to vascular cognitive impairment and mixed dementia.

In addition, IR is associated with a chronic inflammatory state, which may further promote proinflammatory mediator release, oxidative stress, and immune cell infiltration, all of which contribute to BBB injury (Ponce-Lopez, 2025). The IR and metabolic abnormalities reflected by an elevated TyG index may weaken BBB integrity, allowing harmful circulating substances, such as inflammatory mediators and metabolic byproducts, to enter the brain more readily and induce neuronal injury. At the same time, BBB dysfunction may impair the clearance of metabolic waste products such as amyloid-β (Aβ), thereby creating a vicious cycle (Sweeney et al., 2018; Ponce-Lopez, 2025). One study found that, in patients with AD, a higher TyG index was associated with increased BBB permeability (Padovani et al., 2025). These findings suggest that TyG-related metabolic dysfunction may be linked to cognitive impairment, at least in part, through endothelial dysfunction, cerebral blood flow dysregulation, BBB disruption, and cerebral small vessel disease.

### 5.3 Abnormal amyloid-(metabolism and tau pathology

One of the hallmark pathological features of AD is the abnormal accumulation of Aβ in the brain (Abyadeh et al., 2024). Recent evidence suggests that IR may contribute to dysregulated Aβ metabolism and thereby increase the risk of AD (Woodfield et al., 2022). Insulin and insulin-related enzymes, such as insulin-degrading enzyme (IDE), are involved in the degradation and clearance of Aβ (Fu et al., 2020; Mett et al., 2021). Under IR conditions, hyperinsulinemia may competitively inhibit IDE-mediated degradation of Aβ, thereby promoting Aβ accumulation in the brain (Stargardt et al., 2013; Portelius et al., 2017). In addition, IR may impair Aβ clearance by affecting transport mechanisms across the BBB (De Felice et al., 2022; Jeong et al., 2022). Excessive cerebral accumulation of Aβ may lead to the formation of oligomers and plaques, which can trigger synaptic toxicity and neuroinflammation, ultimately resulting in cognitive decline (Kim et al., 2022).

Beyond Aβ metabolism, IR may also contribute to tau pathology. Under normal conditions, insulin signaling through the PI3K/Akt pathway helps inhibit glycogen synthase kinase-3β (GSK-3β), a key kinase involved in tau phosphorylation (Mullins et al., 2017). When insulin signaling is impaired, reduced Akt activity may lead to disinhibition of GSK-3β, thereby promoting tau hyperphosphorylation (Yarchoan et al., 2014; Mullins et al., 2017). Hyperphosphorylated tau can dissociate from microtubules, disrupt axonal transport, impair synaptic function, and aggregate into neurofibrillary tangles. These tau-related changes are strongly associated with neuronal dysfunction and cognitive decline.

Aβ and tau pathology may also interact with insulin signaling abnormalities. Aβ oligomers can impair insulin receptor signaling and promote abnormal IRS-1 phosphorylation, thereby further aggravating brain insulin resistance (Talbot et al., 2012; Mullins et al., 2017). Tau pathology may worsen mitochondrial transport and synaptic function, increasing neuronal vulnerability to metabolic stress (De Felice and Ferreira, 2014; Mullins et al., 2017). Therefore, the IR reflected by an elevated TyG index may be involved in AD-related pathological processes by interfering with normal Aβ metabolism and facilitating tau hyperphosphorylation, which may partly explain its association with progression from MCI to AD.

### 5.4 Neuroinflammatory responses

Chronic low-grade inflammation is an important feature of metabolic syndrome and IR and is also considered to be involved in the onset and progression of neurodegenerative disease (Shimobayashi et al., 2018; Püschel et al., 2022; Fołta et al., 2025). Under IR conditions, adipose tissue releases increased levels of inflammatory mediators, leading to a state of systemic inflammation (Lackey and Olefsky, 2016; McLaughlin et al., 2017). These inflammatory factors may enter the brain through a compromised BBB, activate microglia and astrocytes, and trigger neuroinflammatory responses (Yang et al., 2022). Activated microglia can release large amounts of proinflammatory cytokines and reactive oxygen species, further damaging neurons and synapses (Wang H. K. et al., 2023).

In addition, IR may directly activate inflammatory signaling pathways in the brain, such as the nuclear factor-κB (NF-κB) pathway, thereby increasing the production of inflammatory mediators (Atar and Şahin Anılgan, 2026). Inflammatory signaling may also impair insulin signaling through mechanisms such as c-Jun N-terminal kinase activation and IRS serine phosphorylation, creating a vicious cycle in which inflammation worsens IR and IR further amplifies inflammatory responses (De Felice and Ferreira, 2014). Neuroinflammation may also interact with Aβ and tau pathology. Aβ aggregates can activate microglia and induce inflammatory responses, while chronic inflammation may promote Aβ production, reduce Aβ clearance, and facilitate tau phosphorylation (De Felice and Ferreira, 2014; Mullins et al., 2017).

One study found that an elevated TyG index was associated with cognitive impairment in patients with major depression and that interleukin-6 (IL-6) and tumor necrosis factor-α (TNF-α) partially mediated the association between the TyG index and cognitive scores (Yuan et al., 2026). These findings suggest that the metabolic abnormalities reflected by an elevated TyG index may be linked to cognitive impairment partly through neuroinflammatory pathways. Therefore, neuroinflammatory responses may serve as a key bridge connecting systemic metabolic dysfunction, BBB disruption, AD-related proteinopathy, and cognitive decline.

### 5.5 Integrated model of tyG-related mechanisms in MCI and dementia

It should be noted that the mechanisms described above do not operate in isolation but rather interact with and amplify one another. IR, impaired cerebral energy metabolism, vascular endothelial dysfunction, BBB disruption, Aβ deposition, tau pathology, and neuroinflammation may form a complex pathological network in the development and progression of cognitive impairment. The metabolic disturbances reflected by an elevated TyG index may be involved in, or exacerbate, these pathological processes.

At a holistic level, an elevated TyG index reflects a systemic metabolic phenotype characterized by impaired glucose regulation, hypertriglyceridemia, IR, lipotoxicity, oxidative stress, and low-grade inflammation. In neurons, this metabolic state may impair insulin receptor signaling, reduce glucose utilization, disrupt mitochondrial function, and weaken synaptic plasticity. In the cerebral vasculature, IR may impair the PI3K/Akt/eNOS pathway, reduce NO production, induce endothelial dysfunction, disturb cerebral blood flow regulation, and promote BBB disruption and cerebral small vessel disease. These vascular and metabolic abnormalities may further impair Aβ clearance and promote tau hyperphosphorylation. Meanwhile, Aβ accumulation, tau pathology, oxidative stress, and BBB leakage can activate microglia and astrocytes, thereby amplifying neuroinflammation.

This multisystem interaction may represent an important biological basis for the association between the TyG index and cognitive impairment. Therefore, an elevated TyG index may be associated with cognitive decline and dementia through the combined involvement of multiple interconnected pathways, including metabolic dysfunction, vascular injury, BBB disruption, AD-related proteinopathy, and neuroinflammation. However, these mechanisms remain largely inferential, and further experimental, neuroimaging, biomarker-based, and longitudinal studies are needed to clarify the causal relevance of TyG-related metabolic dysfunction in cognitive decline.

## 6 Clinical utility and potential applications of the TyG index in cognitive impairment

As a simple and readily available metabolic indicator, the TyG index has potential clinical relevance in the field of cognitive impairment, although its clinical utility remains to be fully established.

First, the TyG index may serve as a potential metabolic risk-stratification marker for cognitive risk in primary care and community-based settings. In individuals with elevated TyG levels, the presence of underlying metabolic abnormalities may indicate a higher risk of cognitive decline. In such cases, closer cognitive assessment and follow-up management may be warranted, which could improve the efficiency of early identification and provide a valuable window for timely intervention.

Second, the TyG index may be useful as a dynamic indicator for monitoring metabolic status. Because TyG levels can be influenced by physical activity, diet, and pharmacological treatment, the index may be used to assess changes in metabolic status over time in populations at risk of cognitive impairment. In this context, TyG may serve as an accessible marker for evaluating the effectiveness of metabolic management within multidomain intervention strategies. It should be emphasized, however, that current evidence remains insufficient to support the conclusion that lowering the TyG index directly improves cognitive outcomes. Nevertheless, changes in the TyG index may reflect broader improvements in the systemic metabolic environment.

Third, the TyG index highlights the importance of metabolic factors in cognitive assessment. The development and progression of cognitive impairment involve complex multisystem interactions in which metabolic abnormalities, vascular injury, BBB dysfunction, and neuroinflammation mutually influence one another. The clinical significance of the TyG index lies in reinforcing the role of metabolic factors in the evaluation of cognitive impairment and promoting a shift from a solely neuropathological perspective toward a more integrated, multisystem management approach. In older adults with cognitive impairment or those at high risk, an elevated TyG index may suggest that, in addition to cognitive interventions, metabolic control should also be strengthened.

Overall, the TyG index should currently be regarded as a potential metabolic risk-stratification marker rather than an established diagnostic or predictive biomarker for cognitive impairment. Its main value may lie in identifying individuals with metabolic dysfunction who could benefit from closer cognitive monitoring and integrated vascular-metabolic risk management. However, before incorporation into routine cognitive risk assessment, further studies are needed to validate its incremental predictive value beyond established dementia risk factors, including age, education, APOE ε4 status, hypertension, diabetes, obesity, cardiovascular disease, and lifestyle factors. Future research should also assess discrimination, calibration, reclassification performance, decision-curve utility, and clinically meaningful cutoff values across different populations.

## 7 Limitations of current evidence and future research directions

Although substantial progress has been made in understanding the relationship between the TyG index and cognitive impairment, several important limitations remain and should be addressed in future research. At the same time, as evidence in this field continues to accumulate, several promising directions for further investigation have also emerged.

First, most studies examining the association between the TyG index and cognitive impairment have adopted a cross-sectional design and therefore lack long-term follow-up data. Cross-sectional studies can only provide information on associations between the TyG index and cognitive function but cannot establish temporal or causal relationships. For example, an elevated TyG index may coexist with cognitive impairment without necessarily indicating that TyG elevation contributes to its development. To determine whether the TyG index is independently associated with future cognitive impairment, more prospective cohort studies are needed to track the relationship between baseline TyG levels and subsequent cognitive changes over time. Future studies may also make greater use of existing large-scale cohort databases with available fasting glucose, triglyceride, metabolic, vascular, and cognitive data. For example, cohorts such as the Chronic Renal Insufficiency Cohort (CRIC) and SPRINT/SPRINT-MIND may provide valuable opportunities to examine whether baseline TyG levels or longitudinal changes in TyG are associated with long-term risk of MCI, dementia, or cognitive decline in high-risk populations.

Second, substantial heterogeneity and residual confounding remain important limitations of the available evidence. Many studies have focused on specific populations, such as patients with cardiovascular disease, chronic kidney disease, or diabetes, whereas evidence from the general older population remains relatively limited. In addition, differences in cognitive assessment tools, TyG calculation methods, cutoff values, follow-up durations, and covariate adjustment strategies may affect the comparability and consistency of findings. Cognitive impairment and dementia are influenced by multiple demographic, socioeconomic, vascular, metabolic, lifestyle, and genetic factors, including age, sex, education level, socioeconomic status, adiposity, diabetes, hypertension, dyslipidemia, smoking, alcohol consumption, physical activity, depression, kidney function, cardiovascular disease, stroke history, medication use, and APOE ε4 status (Livingston et al., 2024). Hypertension is particularly important because it is a well-established risk factor for MCI and dementia and frequently coexists with insulin resistance and metabolic dysfunction. Although several studies adjusted for demographic and vascular risk factors, the covariates included in multivariable models varied substantially across studies. Therefore, residual confounding cannot be excluded, and the independent contribution of the TyG index beyond established vascular and metabolic risk factors remains to be fully clarified.

In addition, the available evidence should be interpreted in light of heterogeneity across study designs, populations, cognitive assessment tools, TyG calculation methods, cutoff values, follow-up durations, and adjustment strategies. Some studies have reported positive associations between the TyG index and MCI, dementia, or cognitive decline, whereas others have shown weaker, subgroup-specific, non-linear, or non-significant associations. These inconsistencies may be partly explained by differences in diabetes status, kidney disease, cardiovascular comorbidities, age distribution, dementia subtype, cognitive domain assessed, and the degree to which confounding factors were controlled. Future studies should use harmonized cognitive outcomes, standardized TyG definitions, and consistent covariate adjustment strategies. Direct comparisons between minimally adjusted and fully adjusted models, stratified analyses by hypertension and other vascular risk factors, and sensitivity analyses excluding participants with major cardiovascular or cerebrovascular disease would help clarify whether the TyG index is independently associated with cognitive outcomes.

Third, most current studies have not sufficiently considered the influence of genetic susceptibility factors, particularly the apolipoprotein E (APOE) ε4 genotype, on the relationship between the TyG index and cognition. APOE ε4 is the strongest genetic risk factor for AD and may modify the effects of metabolic dysfunction on cognitive outcomes (Contreras et al., 2026). Future studies should therefore incorporate genetic information and examine potential interactions between the TyG index, APOE ε4 status, and other polygenic risk profiles. Such analyses may help determine whether individuals with both elevated TyG levels and genetic susceptibility are at particularly high risk of cognitive decline or AD-related pathological changes.

Fourth, the TyG index itself has inherent limitations. For example, it cannot distinguish between different mechanisms of IR, such as hepatic IR versus peripheral IR, nor can it directly reflect insulin sensitivity within the central nervous system. In addition, direct evidence comparing the predictive value of the TyG index with fasting triglycerides or fasting glucose alone for cognitive outcomes remains limited. Future studies should determine whether the TyG index provides incremental predictive value over its individual components using direct comparative models. These limitations suggest that TyG findings should be interpreted with caution and evaluated in combination with other relevant indicators.

Accordingly, future research should focus on several key areas. First, multicenter prospective cohort studies should be conducted to validate the general applicability of the TyG index across different ethnicities and geographic regions. Such studies should include diverse populations, including community-dwelling older adults as well as high-risk groups such as patients with cardiovascular disease, chronic kidney disease, and diabetes, and should incorporate long-term follow-up of cognitive outcomes. In addition to newly established cohorts, secondary analyses of existing cohort databases, such as CRIC and SPRINT/SPRINT-MIND, may be useful for evaluating whether baseline TyG index is associated with subsequent MCI or dementia and whether TyG trajectories are associated with the rate of cognitive decline. Large-scale cohort studies would allow for more precise estimation of the effect size of the association between the TyG index and cognitive impairment and may help determine appropriate cutoff values for different populations.

Second, more basic and translational studies are needed to clarify how the TyG index may contribute to neuronal injury and cognitive decline at the mechanistic level. In particular, future work may explore potential causal links between the TyG index and AD-related pathology, including amyloid-β deposition and tau hyperphosphorylation. Future studies should also consider whether APOE ε4 status modifies these associations, given its close relationship with AD risk, lipid metabolism, amyloid pathology, and cerebral glucose metabolism.

Third, direct interventional studies targeting TyG-related metabolic abnormalities remain largely absent. Future studies should therefore employ randomized controlled trial designs to evaluate whether interventions that improve TyG-related metabolic parameters are associated with slower cognitive decline, reduced incidence of MCI or dementia, or favorable changes in neuroimaging and AD-related biomarkers. Possible interventions may include lifestyle modification, such as dietary control, weight management, and physical exercise, as well as pharmacological approaches, including insulin sensitizers and glucagon-like peptide-1 receptor agonists. Importantly, such studies should not only assess whether the TyG index can be reduced but also whether changes in TyG are accompanied by improvements in cognitive trajectories, neuroimaging markers, or AD-related biomarkers. This would help determine whether the TyG index is merely a risk marker or a potentially modifiable target within cognitive prevention strategies.

Fourth, further evaluation of clinical feasibility and utility is warranted. Studies should investigate clinicians’ and patients’ attitudes toward incorporating the TyG index into cognitive assessment, as well as the practical workflow and barriers to its implementation in primary care settings. In parallel, health economic analyses are needed to compare the cost-effectiveness of TyG-based screening strategies with that of conventional screening approaches. Such evidence would help policymakers and healthcare institutions determine whether TyG testing should be incorporated into routine health examinations or comprehensive geriatric assessment.

In summary, many important questions regarding the relationship between the TyG index and cognitive impairment remain unresolved. Future research should continue to advance across multiple dimensions, including prospective cohort validation, genetic stratification, mechanistic investigation, intervention, and real-world application. As evidence continues to grow, a more comprehensive understanding of the role of the TyG index in the development and progression of cognitive impairment may be achieved, thereby informing its potential future use in the early identification of high-risk individuals and in integrated cognitive risk management strategies.

## 8 Conclusion

Current evidence suggests that the TyG index is associated with mild cognitive impairment, dementia, and related pathological changes, possibly through insulin resistance, impaired cerebral energy metabolism, blood–brain barrier disruption, amyloid-β dysregulation, and neuroinflammation. As a simple and accessible metabolic marker, the TyG index may help identify individuals at increased risk of cognitive impairment, although its role as an independent predictive biomarker remains to be fully established. Given the predominance of observational evidence, further prospective, mechanistic, and interventional studies are needed to clarify its causal relevance, incremental predictive value, and clinical utility.
