# Supplementary material for: TyG index and mild cognitive impairment and dementia: a review of current evidence
Source: Front Aging Neurosci. 2026 Jul 22;18:1825259. doi: 10.3389/fnagi.2026.1825259 (PMC13437684; doi:10.3389/fnagi.2026.1825259)
Supplement: Supplementary file 1 [file Data_Sheet_1.pdf]

No.	Citation	Study design	Population / data source	Sample size	TyG-related exposure	Cognitive outcome	Main findings
1	Yuan et al. (2026)(Yuan et al., 2026a)	Cross-sectional study	comorbid major depressive disorder and type 2 diabetes	256	TyG index	Cognitive function	Higher TyG index was statistically associated with cognitive impairment in comorbid major depressive disorder and type 2 diabetes mellitus, partly mediated by peripheral inflammation.
2	Xing et al. (2026)(Xing et al., 2026)	Cross-sectional and longitudinal multi-cohort study	Chongming Older People Screening Project; CHARLS; ELSA	2326/ 3392/ 1825	TyG-WWI	MCI	Sarcopenic obesity defined by sarcopenia combined with elevated TyG-WWI was consistently associated with higher MCI risk across three cohorts. TyG-WWI showed the best performance among obesity indicators for identifying MCI, and the TyG-WWI-based machine learning model demonstrated good discrimination in both internal and external validation.
3	Chen et al. (2026)(Chen et al., 2026b)	Cross-sectional and longitudinal multi-cohort study	Hospital; China Health and Retirement Longitudinal Study	1580/ 4575	C-reactive protein-triglyceride-glucose index(CTI)	MCI; Cognitive impairment; Cognitive function/decline	In both the main cohort and the CHARLS cohort , a high CTI score was independently associated with an increased risk of cognitive impairment. In the main cohort, the CTI significantly outperformed the TyG index in terms of risk reclassification.
4	Jiang et al. (2026)(Jiang et al., 2026)	Prospective cohort study	middle-aged and older Chinese adults	1492	TyG-BMI	Cognitive impairment	Higher AIP and TyG-BMI were associated with a reduced short-term risk of cognitive impairment
5	Mossavarali et al. (2026)(Mossa	Cross-sectional study	Bushehr Elderly Health (BEH) program	2426	TyG index	Cognitive function	TyG index was not significantly associated with cognition.

	varali et al., 2026)						
6	Liu et al. (2026)(Liu et al., 2026)	Retrospective database study	MIMIC-IV | ICU patients with cerebrovascular disease	1754	TyG index	Vascular dementia	Higher TyG levels were independently associated with increased odds of VaD in patients with CVD
7	Wei et al. (2026)(Wei et al., 2026)	Cross-sectional study	Chinese population	876	TyG-BMI; TyG-WC; TyG-WHtR; TyG-WWI; TyG-ABSI; TyG index	Cognitive function	Elevated TyG and its obesity-related indices, particularly TyG-WHtR, are associated with poorer cognitive function in healthy middle-aged Chinese adults.
8	Zhao et al. (2026)(Zhao et al., 2026)	Cross-sectional study	coronary heart disease patients	839	TyG index	MCI; Cognitive impairment; Cognitive function/decline	In patients with CHD, the TyG index was associated with MCI risk, and a potential threshold effect was observed.
9	Yuan et al. (2026)(Yuan et al., 2026b)	Cross-sectional study	major depressive disorder and healthy controls	210/120	TyG index	Cognitive function	Elevated TyG index is associated with poorer cognition in MDD
10	Ahn et al. (2026)(Ahn et al., 2026)	Cross-sectional study	Dementia Platform Korea Trial-Ready Registry	507	TyG index	Cognitive function	Higher TyG index was associated with lower MMSE and delayed recall scores, while no clear association was observed with amyloid PET SUVR.
11	Xu et al. (2026)(Xu et al., 2026)	Cross-sectional study	Acute ischemic stroke	235	TyG index	Cognitive impairment	Elevated TyG index and non-HDL-C were independently associated with early cognitive impairment after acute ischemic stroke.

12	Low et al. (2026)(Low et al., 2026)	Prospective cohort study	type 2 diabetes	1412	TyG-BRI	Cognitive function	TyG-BRI was associated with changes in cognitive function among older adults with T2DM.
13	Liang et al. (2026)(Liang et al., 2026b)	Cross-sectional analyses; Longitudinal cohort study	NHANES; CHARLS	3813/3061	TyG-ABSI; TyG index	Cognitive impairment	The TyG-ABSI index shows a stronger association with cognitive impairment risk and is more effective in identifying at-risk individuals compared to other TyG-derived indices
14	Duan et al. (2026)(Duan et al., 2026)	Prospective cohort study	UK Biobank	356,454	TyG-BMI; TyG-WC; modified TyG indices	Vascular dementia; Alzheimer's disease; Dementia; Brain structure/neuroimaging	Modified TyG indices show nonlinear, differential associations with AD and VaD risks, and are linked to structural brain alterations.
15	Liang et al. (2026)(Liang et al., 2026a)	Cross-sectional study	Parkinson's Disease	251	TyG; TyG-BMI; AIP; METS-IR	MCI; Dementia; Cognitive impairment; Cognitive function/decline; Parkinson's disease cognition	Glucolipotoxicity-based indices (TyG, AIP), unlike BMI-dependent markers (TyG-BMI, METS-IR), are robustly linked to PD dementia through domain-specific linear or nonlinear patterns.
16	Chen et al. (2026)(Chen et al., 2026a)	Cross-sectional study	outpatient	1162	TyG index	Cognitive function	ApoE ε4 carriers demonstrate a distinct metabolic profile characterized by lower BMI and elevated TyG index, associated with poorer cognitive performance.

17	Ge et al. (2026)(Ge et al., 2026)	Prospective cohort study	Atherosclerosis Risk in Communities Study	12601	TyG index	Dementia	A higher level of average TyG index and increase in TyG index were both associated with a higher risk of dementia.
18	Fan et al. (2026)(Fan et al., 2026)	post-hoc trial analysis	a post hoc analysis of the SPRINT (Systolic Blood Pressure Intervention Trial)	7427	TyG index	Probable dementia; cognitive function; white matter hyperintensities	In patients with hypertension with TyG >9.07, lowering blood pressure to <120 mm Hg reduced dementia incidence more than standard control.
19	Yang et al. (2026)(Yang et al., 2026)	Retrospective cohort study	China Health and Retirement Longitudinal Study	5464	CTI; TyG index	Cognitive function	Elevated CTI is independently associated with increased risk of cognitive decline.
20	Zuliani et al. (2025)(Zuliani et al., 2025)	Cross-sectional study	Italian Memory Clinic	1350	TyG index	Cognitive impairment	Insulin resistance may be a risk factor for dementia with a cerebrovascular component, but only in older females.
21	Feng et al. (2025)(Feng et al., 2025c)	Cross-sectional study	type 2 diabetes mellitus patients with and without diabetic kidney disease	243	TyG index	Cognitive function	Elevated TyG index levels have been linked to an increased likelihood of MCI, particularly affecting executive function and immediate recall, among individuals with T2DM but without DKD.
22	Feng et al. (2025)(Feng et al., 2025b)	Cross-sectional observational study	Coronary artery disease	1163	TyG index	Cognitive function	The TyG index is independently associated with cognitive function in patients with Coronary artery disease, with elevated values associated with an increased risk of MCI.

23	Wei et al. (2025)(Wei et al., 2025)	Cross-sectional study; Cohort study	CHARLS; participants aged ⩾45 years from the CHARLS	5657/637	TyG index; TyG-BMI; TyG-WC; TyG-WHtR	Motoric cognitive risk syndrome	TyG-BMI demonstrates a nonlinear association with MCR risk and may serve as an accessible biomarker for early cognitive risk detection in depressive individuals.
24	Cheng et al. (2025)(Cheng et al., 2025)	Cross-sectional study	Parkinson's disease	78	TyG index	Cognitive function	Elevated TyG levels were associated with an increased likelihood of PDCI in patients with PD.
25	Cao et al. (2025)(Cao et al., 2025)	Cross-sectional study; Cohort study	Parkinson's disease	348	TyG index	Cognitive function	TyG is associated with lower cognitive function and higher risk of dementia.
26	Guan et al. (2025)(Guan et al., 2025)	Prospective cohort study	Framingham Heart Study	2170	TyG index	Alzheimer's disease	Higher TyG index trajectories were associated with an increased risk of incident AD.
27	Wu et al. (2025)(Wu et al., 2025)	Cross-sectional study	Atrial fibrillation	142	TyG index	Cognitive function	In patients with AF, the TyG index was found to be unrelated to low cognitive function as assessed by a comprehensive battery of tests.
28	Feng et al. (2025)(Feng et al., 2025a)	Retrospective cohort study	type 2 diabetes	597	TyG index	MCI; Cognitive impairment	Elevated TyG index is associated with the risk of mild cognitive impairment in elderly patients with type 2 diabetes.
29	Ding et al. (2025)(Ding et al., 2025)	Cohort study	National Health and Nutrition Examination Survey	1352	TyG-BMI; TyG index	Cognitive function	The TyG index was negatively associated with cognitive function scores.

			(NHANES)				
30	Hao et al. (2024)(Hao et al., 2024)	Cross-sectional study	Chinese population	1125	TyG-WC; TyG-WHtR; TyG index; TyG-BMI	Cognitive function	There was a nonlinear U-shaped relationship between TyG-WC and cognitive function.
31	Cui et al. (2024)(Cui et al., 2024)	Cohort study	CHARLS	4027	TyG-BMI; METS-IR; TyG index; TG/HDL-C	Cognitive impairment	Elevated levels of four indices are positively correlated with MCI risk.
32	Bai et al. (2024)(Bai et al., 2024)	Prospective cohort study	CHARLS	2959	TyG index	Cognitive impairment;	The study highlights a significant connection between elevated TyG index levels and cognitive impairment among older adults in China.
33	Zhang et al. (2024)(Zhang et al., 2024)	Longitudinal cohort study	non-demented participants	855	TyG-BMI	Cognitive function; AD biomarkers; Brain structure	Higher TyG-BMI was associated with better cognitive / brain structural indicators and lower AD risk.
34	Hou et al. (2024)(Hou et al., 2024)	Longitudinal cohort study	CHARLS	8946	TyG-WHtR	Cognitive impairment	Higher TyG-WHtR was associated with an increased risk of cognitive impairment among middle-aged and older adults.
35	Ma et al. (2024)(Ma et al., 2024)	Cross-sectional study	Community population	1484	TyG index	Cognitive function	The present study suggested that an elevated TyG index was associated with a higher CI risk.
36	Tian et al. (2024)(Tian et al., 2024)	Cross-sectional study	Community population	4541	TyG index	Cognitive function; Brain structure	Insulin resistance, as indicated by a high TyG index, was associated with poor function in multiple cognitive domains and global brain atrophy.

37	Cheng et al. (2024)(Cheng et al., 2024)	Prospective cohort study	Acute ischemic stroke	313	TyG index	Cognitive function	A higher TyG index level at admission was independently correlated with increased risk of Post-stroke cognitive impairment three months later and could be used as a predictor.
38	Sun et al. (2023)(Sun et al., 2023)	Prospective cohort study	Framingham Heart Study	2170	TyG index	Alzheimer's disease	This study showed that moderately elevated TyG index was independently associated with a higher incidence of Alzheimer's disease.
39	Wei et al. (2023)(Wei et al., 2023)	Cohort study	NHANES	661	TyG index	Cognitive function	A high TyG index was strongly correlated with low cognitive function evaluated through the CERAD test (CERAD-WL and CERAD-DR) among US nondiabetic older women.
40	Tian et al. (2023)(Tian et al., 2023)	Population-based cross-sectional study	Multimodal Interventions to Delay Dementia and Disability in rural China	5199	TyG index	Vascular dementia; Alzheimer's disease; Dementia; AD biomarkers	High TyG index was associated with higher likelihoods of dementia and AD, and was correlated with elevated plasma Aβ but not total tau or NfL.
41	Li et al. (2022)(Li et al., 2022)	cohort study	Middle-Aged to Elderly Population	1774	TyG index	Cognitive function	This study demonstrated that increasing values of the TyG index were positively associated with the risk of cognitive decline.
42	Tong et al. (2022)(Tong et al., 2022)	Cross-sectional study	T2DM	517	TyG-BMI; TyG index	Cognitive function	TyG index and TyG-BMI are associated with MCI in T2D patients.
43	Chen et al. (2022)(Chen et al., 2022)	Prospective cohort study	ELSA	1742	TyG index	Cognitive function	CRP and TyG significantly mediated the association between lung function and subsequent cognitive function.

44	Wang et al. (2022)(Wang et al., 2022)	Longitudinal cohort study	CHARLS	4420	TyG index	Cognitive function	The increased TyG index is related to the risk of cognitive decline in men.
45	Teng et al. (2022)(Teng et al., 2022)	Retrospective study	T2DM	308	TyG index	Cognitive function	Higher TyG index was associated with increased risks of cognitive impairment and severe CSVD burden in elderly patients with T2DM, with CSVD burden partly mediating this association.
46	Huang et al. (2022)(Huang et al., 2022)	Cross-sectional study	Taiwan Biobank	28486	TyG index	Cognitive function	TyG index increased with worsening cognitive impairment but was not clearly identified as an independent predictor in the multivariable model.
47	Weyman-Vela et al. (2022)(Weyman-Vela et al., 2022)	Case-control study	Older adults with and without MCI	65/70	TyG index	Cognitive function	The TyG index is independently associated with the presence of MCI in older people.
48	Hong et al. (2021)(Hong et al., 2021)	Retrospective cohort study	National Health Information Database	5586048	TyG index	Dementia; Alzheimer's disease; vascular dementia	TyG index was associated with an increased risk of dementia, including AD and VD
49	Jiang et al. (2021)(Jiang et al., 2021)	Cross-sectional study	Cerebral Small Vessel Disease	280	TyG index	Vascular cognitive impairment; cognitive function	TyG index is associated with vascular cognitive impairment in patients with cerebral small vessel disease.

Reference:

1 Ahn, Y.H., Kang, J.G., Choi, D., Kim, D.J., Yang, C.M., Lee, S.Y., et al. (2026). Liver-metabolic stress, apolipoprotein E ε4, and cognition and amyloid burden: findings from the dementia platform Korea trial-ready registry. *Front Aging Neurosci* 18**,** 1773977. doi: 10.3389/fnagi.2026.1773977.

2 Bai, W., An, S., Jia, H., Xu, J., and Qin, L. (2024). Relationship between triglyceride-glucose index and cognitive function among community-dwelling older adults: a population-based cohort study. *Front Endocrinol (Lausanne)* 15**,** 1398235. doi: 10.3389/fendo.2024.1398235.

3 Cao, H., Zhao, Y., Chen, Z., Zou, X., Du, X., Yi, L., et al. (2025). Triglyceride-glucose index predicts cognitive decline and striatal dopamine deficiency in Parkinson disease in two cohorts. *NPJ Parkinsons Dis* 11(1)**,** 240. doi: 10.1038/s41531-025-01100-1.

4 Chen, C., Lu, Z., Wang, X., Zhang, J., Zhang, D., and Li, S. (2022). The chain mediating role of C-reactive protein and triglyceride-glucose index between lung function and cognitive function in a systemic low-grade inflammation state. *J Psychiatr Res* 155**,** 380–386. doi: 10.1016/j.jpsychires.2022.09.004.

5 Chen, R., Fan, S., Di, C., Wu, H., Shi, Z., Liu, F., et al. (2026a). Insulin resistance (TyG index) and body mass index as metabolic biomarker combined with ApoE genotype to diagnose Alzheimer's disease. *Front Aging Neurosci* 18**,** 1731547. doi: 10.3389/fnagi.2026.1731547.

6 Chen, Y., Sun, J., Chen, X., Yang, S., Cong, X., Ge, Z., et al. (2026b). A multi-stage validation of the C-reactive protein-triglyceride-glucose index for predicting mild cognitive impairment: evidence from clinical and nationwide prospective cohorts. *Front Endocrinol (Lausanne)* 17**,** 1841431. doi: 10.3389/fendo.2026.1841431.

7 Cheng, Y., You, S., Wang, X., Ge, Y., Li, L., Ren, T., et al. (2025). A high triglyceride-glucose index correlates with cognitive impairment in Parkinson's disease: a cross-sectional study. *Front Neurosci* 19**,** 1620118. doi: 10.3389/fnins.2025.1620118.

8 Cheng, Y., Zhu, H., Sun, D., Li, L., Liu, C., Sun, S., et al. (2024). High triglyceride-glucose index at admission is a predictor of post-stroke cognitive impairment in patients with acute ischemic stroke. *J Stroke Cerebrovasc Dis* 33(1)**,** 107510. doi: 10.1016/j.jstrokecerebrovasdis.2023.107510.

9 Cui, Y., Xu, Z., Cui, Z., Guo, Y., Wu, P., and Zhou, X. (2024). Comparative study of insulin resistance surrogate indices to predict mild cognitive impairment among Chinese non-diabetic adults. *Lipids Health Dis* 23(1)**,** 357. doi: 10.1186/s12944-024-02353-0.

10 Ding, C., Lu, R., Kong, Z., and Huang, R. (2025). Exploring the triglyceride-glucose index's role in depression and cognitive dysfunction: Evidence from NHANES with machine learning support. *J Affect Disord* 374**,** 282–289. doi: 10.1016/j.jad.2025.01.051.

11 Duan, H., Liu, J., Pan, X., Huang, T., and Chen, X. (2026). Associations of modified triglyceride-glucose indices with risks of dementia subtypes and brain structure: a prospective cohort study. *Front Neurol* 17**,** 1750736. doi: 10.3389/fneur.2026.1750736.

12 Fan, C., Wang, X., Tao, L., Liao, Y., Jin, L., Shi, Q., et al. (2026). Insulin Resistance and Blood Pressure Control on Cognitive Outcomes. *Hypertension* 83(1)**,** 199–211. doi: 10.1161/hypertensionaha.125.24666.

13 Feng, J., Teng, Z., and Chen, S. (2025a). Associations of obesity-related indices with mild cognitive impairment in adults 60 years and older with type 2 diabetes: a retrospective study. *PeerJ* 13**,** e19442. doi: 10.7717/peerj.19442.

14 Feng, L., Li, J., Zhao, X., Song, J., Yang, S., Wang, X., et al. (2025b). Triglyceride-glucose index as a novel biomarker for mild cognitive impairment in patients with coronary artery disease. *Biomark Med* 19(18)**,** 877–884. doi: 10.1080/17520363.2025.2570634.

15 Feng, S., Zhang, Z., Gao, Q., Huang, L., Zhang, Y., He, M., et al. (2025c). Different association between triglyceride-glucose index and mild cognitive impairment in type 2 diabetes mellitus patients with and without diabetic kidney disease. *Front Nutr* 12**,** 1681164. doi: 10.3389/fnut.2025.1681164.

16 Ge, T., Ding, Y., Yan, M., Zhao, M., Hailili, G., Wu, M., et al. (2026). Longitudinal changes in triglyceride-glucose index and the risk of incident dementia: a prospective cohort study. *J Gerontol A Biol Sci Med Sci* 81(1). doi: 10.1093/gerona/glaf237.

17 Guan, C.K., Gu, Z.B., Sun, J.Y., Zhu, W.G., and Ye, M. (2025). Relationship between TyG trajectories and Alzheimer's disease: data from the Framingham Heart Study. *Rev Clin Esp (Barc)* 225(7)**,** 502331. doi: 10.1016/j.rceng.2025.502331.

18 Hao, J., Lu, Y., Zhang, L., Li, X., Wen, H., Zhao, X., et al. (2024). Association of triglyceride glucose index combined with obesity indicators with cognitive impairment. *Lipids Health Dis* 23(1)**,** 397. doi: 10.1186/s12944-024-02388-3.

19 Hong, S., Han, K., and Park, C.Y. (2021). The insulin resistance by triglyceride glucose index and risk for dementia: population-based study. *Alzheimers Res Ther* 13(1)**,** 9. doi: 10.1186/s13195-020-00758-4.

20 Hou, D.C., Wei, Y., Sun, Y.M., Pei, L.J., and Chen, G. (2024). [A cohort study of association between triglyceride glucose index-waist to height ratio and cognitive impairment in middle-aged and elderly population in China]. *Zhonghua Liu Xing Bing Xue Za Zhi* 45(6)**,** 802–808. doi: 10.3760/cma.j.cn112338-20231226-00375.

21 Huang, S.H., Chen, S.C., Geng, J.H., Wu, D.W., and Li, C.H. (2022). Metabolic Syndrome and High-Obesity-Related Indices Are Associated with Poor Cognitive Function in a Large Taiwanese Population Study Older than 60 Years. *Nutrients* 14(8). doi: 10.3390/nu14081535.

22 Jiang, T., Zhou, Y., Zhang, D., Cao, Z., Bian, L., Ni, Y., et al. (2021). Association of Serum Interleukin-34 and Insulin Resistance with Cognitive Impairment in Patients with Cerebral Small Vessel Disease. *Curr Neurovasc Res* 18(4)**,** 446–455. doi: 10.2174/1567202618666211110113323.

23 Jiang, Y., Zhu, X., Zhao, L., Du, Y., Wang, H., Fan, H., et al. (2026). The atherogenic index of plasma and triglyceride glucose-body mass index are inversely associated with cognitive impairment in middle-aged and older Chinese adults: A community-based cohort study. *J Alzheimers Dis* 111(2), 581–591. doi: 10.1177/13872877261434985.
24 Li, S., Deng, X., and Zhang, Y. (2022). The Triglyceride-Glucose Index Is Associated with Longitudinal Cognitive Decline in a Middle-Aged to Elderly Population: A Cohort Study. *J Clin Med* 11(23). doi: 10.3390/jcm11237153.
25 Liang, H., Jia, Y., Zhang, H., Wang, D., Yu, H., Yan, Y., et al. (2026a). Insulin Resistance Surrogates and Cognitive Impairment in Parkinson's Disease: A Cross-Sectional Study with Interpretable Machine Learning. *Biomedicines* 14(3). doi: 10.3390/biomedicines14030493.
26 Liang, Z., Su, L., Shi, Y., Cai, G., Wu, W., An, S., et al. (2026b). Triglyceride glucose-a body shape index (TyG-ABSI) as a potential indicator for cognitive impairment: Results from two nationally representative cohort studies. *J Affect Disord* 396, 120809. doi: 10.1016/j.jad.2025.120809.
27 Liu, X., Xu, H., Han, X., and Zhang, Q. (2026). Association between triglyceride-glucose index and vascular dementia in ICU patients with cerebrovascular disease: a retrospective study based on the MIMIC-IV database. *BMC Neurol* 26(1). doi: 10.1186/s12883-026-04915-4.
28 Low, S., Moh, A., Zheng, H., Goh, K.S., Chuan, T.B., Ang, K., et al. (2026). Understanding the role of triglyceride glucose-body roundness index (TyG-BRI) in the paradigm of cognitive decline over time in patients with type 2 diabetes. *J Diabetes Complications* 40(3), 109270. doi: 10.1016/j.jdiacomp.2026.109270.
29 Ma, Y., Wei, S., Dang, L., Gao, L., Shang, S., Hu, N., et al. (2024). Association between the triglyceride-glucose index and cognitive impairment in China: a community population-based cross-sectional study. *Nutr Neurosci* 27(4), 342–352. doi: 10.1080/1028415x.2023.2193765.
30 Mossavarali, S., Mohseni, S., Fahimfar, N., Sajadi, N.A., Pejman Sani, M., Sharifi, F., et al. (2026). Novel anthropometric indices and cognitive function among iranian older adults: Bushehr Elderly Health (BEH) program. *PLoS One* 21(4), e0343425. doi: 10.1371/journal.pone.0343425.
31 Sun, J., Xie, Z., Wu, Y., Liu, X., Ma, J., Dong, Y., et al. (2023). Association of the Triglyceride-Glucose Index With Risk of Alzheimer's Disease: A Prospective Cohort Study. *Am J Prev Med* 65(6), 1042–1049. doi: 10.1016/j.amepre.2023.07.011.
32 Teng, Z., Feng, J., Dong, Y., Xu, J., Jiang, X., Chen, H., et al. (2022). Triglyceride glucose index is associated with cerebral small vessel disease burden and cognitive impairment in elderly patients with type 2 diabetes mellitus. *Front Endocrinol (Lausanne)* 13, 970122. doi: 10.3389/fendo.2022.970122.
33 Tian, N., Fa, W., Dong, Y., Liu, R., Liu, C., Liu, K., et al. (2023). Triglyceride-glucose index, Alzheimer's disease plasma biomarkers, and dementia in older adults: The MIND-China study. *Alzheimers Dement (Amst)* 15(2), e12426. doi: 10.1002/dad2.12426.
34 Tian, N., Song, L., Hou, T., Fa, W., Dong, Y., Liu, R., et al. (2024). Association of Triglyceride-Glucose Index With Cognitive Function and Brain

Atrophy: A Population-Based Study. *Am J Geriatr Psychiatry* 32(2), 151–162. doi: 10.1016/j.jagp.2023.09.007.
35 Tong, X.W., Zhang, Y.T., Yu, Z.W., Pu, S.D., Li, X., Xu, Y.X., et al. (2022). Triglyceride Glucose Index is Related with the Risk of Mild Cognitive Impairment in Type 2 Diabetes. *Diabetes Metab Syndr Obes* 15, 3577–3587. doi: 10.2147/dmso.S389327.
36 Wang, K., Xu, L., Liu, L., Zhan, S., Wang, S., and Song, Y. (2022). Sex differences in the association between the change in triglyceride-glucose index and cognitive decline: A population-based cohort study. *J Affect Disord* 316, 42–49. doi: 10.1016/j.jad.2022.08.014.
37 Wei, B., Dong, Q., Ma, J., and Zhang, A. (2023). The association between triglyceride-glucose index and cognitive function in nondiabetic elderly: NHANES 2011-2014. *Lipids Health Dis* 22(1), 188. doi: 10.1186/s12944-023-01959-0.
38 Wei, J., Wu, X., Chen, L., Wang, Y., Zhao, Y., Zhang, L., et al. (2026). Association of TyG index and obesity indicators with cognitive function: a cross - sectional study from Chinese health check-up centers. *BMC Endocr Disord* 26(1). doi: 10.1186/s12902-026-02280-4.
39 Wei, Z.K., Li, C.Y., Liu, Z.Y., Wang, B.L., Wang, C., Liu, Y., et al. (2025). Nonlinear association between TyG-related Indices and motoric cognitive risk syndrome in depressive individuals: evidence from the CHARLS study. *Front Psychiatry* 16, 1622973. doi: 10.3389/fpsyt.2025.1622973.
40 Weyman-Vela, Y., Simental-Mendía, L.E., Camacho-Luis, A., Gamboa-Gómez, C.I., and Guerrero-Romero, F. (2022). The Triglycerides and Glucose Index Is Associated with Mild Cognitive Impairment in Older Adults. *Endocr Res* 47(2), 89–93. doi: 10.1080/07435800.2022.2061508.
41 Wu, J., Wang, Z., Ye, C., Liu, J., Chen, S., Tao, W., et al. (2025). Association between the triglyceride-glucose index and cognitive function in stroke free patients with atrial fibrillation: A cross-sectional study. *Clin Neurol Neurosurg* 253, 108890. doi: 10.1016/j.clineuro.2025.108890.
42 Xing, Y., Liu, Q., He, X., Ma, L., Song, Y., and Ph, D.N. (2026). Triglyceride-Glucose-Waist-to-Weight Index: Novel Biomarker for Mild Cognitive Impairment in Older People With Sarcopenic Obesity. *J Cachexia Sarcopenia Muscle* 17(3), e70307. doi: 10.1002/jcsm.70307.
43 Xu, J., Zhu, S., Wang, T., and Lu, W. (2026). Combined value of triglyceride-glucose index and non-high-density lipoprotein cholesterol in predicting early cognitive impairment after acute ischemic stroke. *Front Neurol* 17, 1713324. doi: 10.3389/fneur.2026.1713324.
44 Yang, L., Zeng, J., Li, L., and Zhang, Y. (2026). C-reactive protein-triglyceride glucose index and risk of cognitive decline in Chinese middle-aged and older adults: A 9-year national cohort study from CHARLS. *J Alzheimers Dis* 109(1), 352–361. doi: 10.1177/13872877251393522.
45 Yuan, Q., He, S., Elhassan, M.A.M., Yu, C., Lu, J., Fang, L., et al. (2026a). Peripheral inflammation mediates the association between triglyceride-glucose index and cognitive impairment in comorbid major depressive disorder and type 2 diabetes: Translational implications for Alzheimer's disease risk via neuroinflammatory pathways. *J Affect Disord* 407, 121830. doi: 10.1016/j.jad.2026.121830.
46 Yuan, Q., Wang, L., Zhu, X., Wu, Z., He, S., Zhang, H., et al. (2026b). Metabolic dysfunction and cognitive impairment in patients with depression: The role of the TyG index and peripheral inflammation. *J Affect Disord* 397, 121026. doi: 10.1016/j.jad.2025.121026.

47 Zhang, Z., Chen, X., and Sheng, Z. (2024). Association of triglyceride glucose-body mass index with Alzheimer's disease pathology, cognition and brain structure in non-demented people. *Sci Rep* 14(1), 16097. doi: 10.1038/s41598-024-67052-3.

48 Zhao, S., Zhao, Y., Wu, X., Li, C., and Peng, H. (2026). Threshold effects of insulin resistance on cognitive function in coronary heart disease: the non-linear diagnostic value of the triglyceride-glucose index. *Front Cardiovasc Med* 13, 1785441. doi: 10.3389/fcvm.2026.1785441.

49 Zuliani, G., Brombo, G., Dario, F.D.P., Zuin, M., Romagnoli, T., Polastri, M., et al. (2025). Sex-difference in the association between Triglyceride-Glucose (TyG) index and dementia. *J Diabetes Metab Disord* 24(2), 231. doi: 10.1007/s40200-025-01744-z.
